# States With Highest and Lowest Cardiovascular Disease‐Related Mortality in the United States (1999−2019): Top and Bottom 3

**DOI:** 10.1002/clc.70256

**Published:** 2026-01-16

**Authors:** Muhammad Umer Sohail, Ruqiat Masooma Batool, Muhammad Saad, Saad Ahmed Waqas, Asad Ali Ahmed Cheema, Abdul Mannan Khan Minhas

**Affiliations:** ^1^ Department of Medicine Dow University of Health Sciences Karachi Pakistan; ^2^ International School of Medicine International University of Kyrgyzstan Bishkek Kyrgyzstan; ^3^ Section of Cardiology, Department of Medicine Baylor College of Medicine Houston Texas USA

## Abstract

**Background:**

Despite declines since the 1960s, cardiovascular diseases (CVDs) remain the leading cause of mortality in the United States. However, recent data indicate stabilization or increases in certain regions, highlighting persistent disparities. This study analyzes trends in states with the highest and lowest CVD‐related age‐adjusted mortality rates (AAMRs) from 1999 to 2019.

**Methods:**

Using CDC WONDER, we conducted a retrospective analysis of CVD‐related mortality in adults aged ≥ 25 years. AAMRs were calculated using ICD‐10 codes I00‐I99, and trends were assessed using Joinpoint regression for annual percent change (APC) and average annual percent change (AAPC).

**Results:**

Between 1999 and 2019, national AAMR declined from 798.47 to 595.56 per 100 000 (AAPC: −1.5%, 95% CI: −1.8% to −1.2%). Mississippi had the highest AAMR (902.23) with the slowest decline, whereas Arizona had the lowest (530.40) with a steeper reduction. Males (702.15), non‐Hispanic Black individuals (850.32), and nonmetropolitan populations (645.21) had persistently higher mortality. Urban‐rural disparities widened over time.

**Conclusion:**

State‐level variations in CVD mortality reflect persistent socioeconomic, behavioral, and healthcare disparities. These findings highlight widening regional gaps and emphasize the need for stronger, state‐specific public health strategies, improved access to preventive care, and targeted interventions for disproportionately affected groups. Strengthening surveillance systems, expanding evidence‐based cardiovascular prevention programs, and addressing structural determinants of health will be essential to reduce the observed disparities and sustain long‐term progress in CVD mortality reduction across the United States.

## Graphical abstract

From 1999 to 2019, US cardiovascular disease mortality declined overall, but progress slowed after 2011 and remained uneven across states. Mississippi, West Virginia, and Oklahoma showed persistently the highest age‐adjusted mortality, with marked sex and racial disparities, especially among non‐Hispanic Black populations.

## 1 Introduction

Cardiovascular diseases (CVDs) have been the leading cause of mortality globally, accounting for approximately one‐third of all global deaths [1]. Despite a significant decline in CVD mortality rates since the 1960s, recent data highlight a concerning trend in mortality rates over the last few years [2, 3].

CVDs impose a substantial economic burden, with annual healthcare expenditures exceeding $320 billion, accounting for approximately 15% of total healthcare spending in the United States [4]. These clinical and economic impacts underscore the importance of continually monitoring mortality trends to identify high‐risk groups and guide effective prevention strategies.

Although geographic disparities in CVD mortality have been documented, yet state‐level trends remain somewhat underexplored despite evidence that some states deviate significantly from national rates [5]. Understanding these differences is critical for informing targeted interventions and health policy approaches.

This study addresses this gap by analyzing CVD‐related mortality trends from 1999 to 2019 across the top three and bottom three states, compared to national mortality rates. The analysis utilizes data from the Centers for Disease Control and Prevention's (CDC) Wide‐Ranging Online Data for Epidemiologic Research (WONDER). This investigation provides insights into regional disparities and contributes to understanding the evolving burden of CVD in the United States.

## 2 Methods

We conducted a retrospective analysis of CVD‐related mortality in the United States from 1999 to 2019. Mortality data were sourced from CDC WONDER [6], encompassing deaths where CVD, identified by ICD‐10 codes I00–I99, was documented as either an underlying or contributing cause. Specifically, we used the Multiple Cause of Death (MCOD) database within CDC WONDER, which includes all death certificates for US residents and provides information on underlying and contributing causes of death. The analysis included individuals aged 25 years and older. Mortality rates were assessed using age‐adjusted mortality rates (AAMRs) per 100 000 individuals (2000 US standard population was used for age‐standardization). Age‐standardization was performed using the direct method, applying age‐specific mortality rates to the 2000 US standard population. Age groups were categorized into standard 10‐year intervals (25–34, 35–44, 45–54, 55–64, 65–74, 75–84, and ≥ 85 years). National AAMRs were determined by aggregating data across 50 states and the District of Columbia. States were ranked based on their AAMRs, and detailed examinations were conducted for the three states with the highest AAMRs (Mississippi, Oklahoma, and West Virginia) and the three states with the lowest AAMRs (Arizona, Minnesota, and Utah). In these states, mortality data were further stratified by sex, race, ethnicity, and urbanization to identify demographic and geographic disparities. Statistical analyses utilized the Joinpoint Regression Program (Version 5.2.0, National Cancer Institute) to compute the annual percent change (APC) and average annual percent change (AAPC), with 95% CIs. A maximum of four joinpoints was allowed. A trend was classified as increasing or decreasing if the slope significantly differed from zero (*p* ≤ 0.05). All statistical tests were two‐tailed, and significance was defined at a *p* ≤ 0.05. A formal test of parallelism was performed within the Joinpoint software to evaluate whether state‐specific trends differed significantly from the national trend. This test compares the slopes of the regression segments across groups, with non‐parallel trends indicating statistically significant differences (*p* ≤ 0.05).

## 3 Results

Between 1999 and 2019, 29 455 193 CVD‐related deaths were recorded among US adults aged 25 years and older (Central Illustration 1) (Table 1). The AAMR declined from 798.47 (797.15 to 799.79) in 1999 to 595.56 (594.61 to 596.50) in 2019, with a decline from 1999 to 2011 (APC: −2.38 [–2.60 to −2.20]) and stability from 2011 to 2019 (APC: −0.13 [−0.47 to 0.31]).

**Central Illustration 1:**
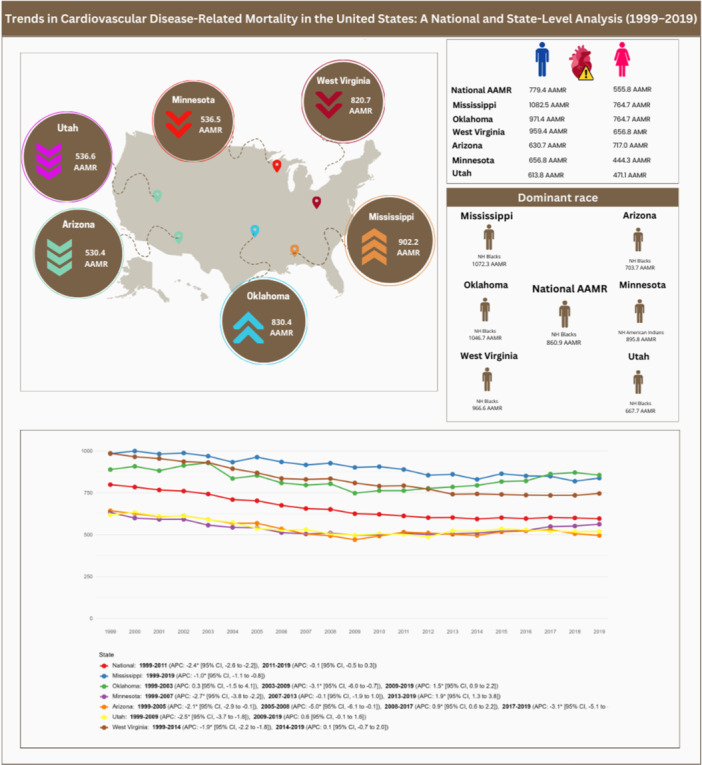
From 1999 to 2019, US cardiovascular mortality declined but slowed after 2011. Marked state disparities persisted: Mississippi, West Virginia, and Oklahoma had the highest AAMR, while Utah, Arizona, and Minnesota were lowest, with notable sex‐ and race‐specific differences.

**Table 1:** Joinpoint regression trends in age‐adjusted cardiovascular disease mortality (AAMR) in the United States, 1999−2019.

State	Trend 1		Trend 2		Trend 3		Trend 4		P for Parallelism (State vs. U.S.)
	Years	APC	Years	APC	Years	APC	Years	APC	
**United States**	1999–2011	−2.4* (−2.6 to −2.2)	2011–2019	−0.1 (−0.5 to 0.3)	—	—	—	—	—
**Mississippi**	1999–2019	−1.0* (−1.1 to −0.8)	—	—	—	—	—	—	0.02
**Oklahoma**	1999–2003	0.3 (−1.5 to 4.1)	2003–2009	−3.1* (−6.0 to −0.7)	2009–2019	1.5* (0.9 to 2.2)	—	—	0.002
**Minnesota**	1999–2007	−2.7* (−3.8 to −2.2)	2007–2013	−0.1 (−1.9 to 1.0)	2013–2019	1.9* (1.3 to 3.8)	—	—	0.003
**Arizona**	1999–2005	−2.1* (−2.9 to −0.1)	2005–2008	−5.0* (−6.1 to −0.1)	2008–2017	0.9* (0.6 to 2.2)	2017–2019	−3.1* (−5.1 to −0.5)	0.004
**Utah**	1999–2009	−2.5* (−3.6 to −1.8)	2009–2019	0.5(−0.05 to 1.6)	—	—	—	—	0.29
**West Virginia**	1999–2014	−1.9*(−2.1 to −1.7)	2014–2019	0.1(−0.6 to 1.9)	—	—	—	—	0.98

*Note:* Trends are shown as Joinpoint‐defined time segments with annual percent change (APC) and 95% confidence intervals (CI). P for parallelism compares each state's trend pattern with the US trend (significant *p* values indicate non‐parallel trends). Asterisk (*): APC is significantly different from zero when *p* < 0.05.

Abbreviations: APC, annual percent change; CI, confidence interval; US, United States.

Mississippi (902.23), Oklahoma (830.40), and West Virginia (820.72) had the highest AAMRs. In Mississippi, rates declined from 1999 to 2019 (APC: −0.99 [−1.13 to −0.84]). Oklahoma's rates were stable from 1999 to 2003 (APC: 0.28 [−1.49 to 4.05]), declined until 2009 (APC: −3.10 [−5.95 to −0.68]), and rose from 2009 to 2019 (APC: 1.50 [0.92 to 2.23]); overall, the long‐term trend was not statistically significant (AAPC: −0.14 [−0.37 to 0.18]). West Virginia experienced a decline in AAMR from 1999 to 2014 (APC: −1.94 [−2.15 to −1.78]), followed by a period of stability until 2019 (APC: 0.13 [−0.67 to 1.96]), yielding an overall significant downward trend (AAPC: −1.42 [−1.57 to −1.30]).

Arizona (530.40), Minnesota (536.45), and Utah (536.60) had the lowest AAMRs. Arizona experienced declines from 1999 to 2005 (APC: −2.13 [−2.93 to −0.12]) and 2005 to 2008 (APC: −4.98 [−6.13 to −0.13]), followed by a rise till 2017 (APC: 0.92 [0.59 to 2.20]) and a decline until 2019 (APC: −3.07 [−5.08 to −0.47]); overall, the long‐term trend demonstrated a significant decline (AAPC: −1.30 [−1.49 to −1.07]). Minnesota saw declines from 1999 to 2007 (APC: −2.69 [−3.80 to −2.22]), stability from 2007 to 2013 (APC: −0.08 [−1.92 to 0.96]), and an increase from 2013 to 2019 (APC: 1.94 [1.28 to 3.80]), resulting in a modest yet significant overall decrease (AAPC: −0.54 [−0.69 to −0.39]). Utah experienced a decline until 2009 (APC: −2.46 [−3.65 to −1.80]), followed by stability until 2019 (APC: 0.57 [−0.05 to 1.62]), corresponding to a significant overall downward trend (AAPC: −0.96 [−1.22 to −0.69]).

## 4 Discussion

Demographic patterns across all states showed similar patterns. Men had higher AAMRs, with values of 779.43 as the national AAMR, 1082.54 in Mississippi, 971.40 in Oklahoma, 959.43 in West Virginia, 656.81 in Minnesota, 630.73 in Arizona, and 613.76 in Utah. Non‐Hispanic (NH) Black or African American individuals had the highest AAMRs nationally (860.40) and across most states, including Mississippi (1072.28), Oklahoma (1046.69), West Virginia (966.55), Arizona (703.67), and Utah (667.71). However, in Minnesota, NH American Indians or Alaskan Natives had the highest AAMR (895.78). Nonmetropolitan areas consistently showed higher mortality rates compared to metropolitan areas, with AAMRs of 726.39 as the national AAMR, 949.52 in Mississippi, 883.75 in Oklahoma, 858.49 in West Virginia, 596.14 in Arizona, 568.10 in Minnesota, and 561.41 in Utah.

In this analysis, we report several important findings. There has been an overall reduction in CVD‐related mortality in the United States from 1999 to 2011 with stabilization of mortality rates from 2011 to 2019. Mississippi, Oklahoma, and West Virginia had the highest AAMRs, whereas Arizona, Minnesota, and Utah had the lowest AAMRs. The NH Black or African American population, males, and nonmetropolitan populations fared worse in all studied states, except in Minnesota, where the NH American Indian or Alaskan Native population had the highest mortality rates.

Mississippi's trends are driven by a confluence of behavioral, socioeconomic, and healthcare‐related factors. The state's high AAMRs are compounded by significant structural challenges, including a poverty rate of 60% which limits access to healthcare and exacerbates health disparities [7]. Behavioral risk factors, such as a higher prevalence of smoking (22.2% vs. 17.1% nationally), lower levels of physical activity (44.7% vs. 50.6%), and a greater proportion of adults who are overweight or obese (69.1% vs. 66.6%), further elevate CVD risk [8]. Mississippi's high intake of sodium, sugars, and fried foods [9], compounded by adverse social determinants of health and unfavorable socio‐economic conditions, significantly contributes to the state's mortality outcomes. Despite these persistent risk factors, Mississippi has demonstrated a gradual but steady decline in CVD‐related mortality rates over the past two decades. Mississippi has seen a 13.9% reduction in adult smoking rates between 2000 (23.8%) and 2018 (20.5%) [10].

Oklahoma faces significant health challenges, including higher smoking rates (20.2% vs. 17.1% nationally), overweight or obesity (70.6% vs. 66.6%), and physical inactivity (42.5% vs. 50.6%) [11]. The state ranks fifth in obesity, with 30.3% of the population affected, and American Indians, who make up 16% of residents, experience elevated rates of obesity (37.7%), diabetes (19.7%), and hypertension (36.8%) [12, 13]. Additionally, Oklahoma has high uninsured rates, with 16% of adults lacking health coverage, and a high poverty rate, both of which further exacerbate cardiovascular health disparities [14]. These insurance gaps reflect broader state‐level policy differences, such as variations in Medicaid expansion and public health funding, which directly influence access to preventive care and chronic disease management.

West Virginia's highest CVD‐related mortality rates can be attributed to significant behavioral and physiological risk factors. These include high adult smoking prevalence (26.9% vs. 17.1% nationally), obesity (71.7% vs. 66.6%), hypertension (33.3%), and high cholesterol (42.4%), coupled with low physical activity (28.8% vs. 50.6%) and poor dietary habits (19.8% consuming adequate fruits/vegetables).

Minnesota consistently reports the lowest CVD mortality rate in the United States, with a 29% lower rate than the national average in 2021 [15]. Minnesota's lower CVD rates are driven by lower smoking prevalence (14.5% vs. 17.1% nationally), higher physical activity (51.1% vs. 50.6% nationally), better healthcare access (91.8% vs. 89.5% nationally), fewer heart disease (3.4% vs. 3.9% nationally) and stroke cases (2.4% vs. 3% nationally), and reduced socioeconomic disparities (Medicaid/CHIP: 14% vs. 19% nationally) [16].

In Arizona, smoking rates (15.6% vs. 17.1%) and overweight or obesity rates (64.8% vs. 66.6%) are lower than the national rates. Whereas Medicaid/CHIP coverage (21% vs. 19%) and physical activity rates (52.7% vs. 50.6%) exceed national rates [17]. Utah's lowest CVD mortality is likely due to lower smoking prevalence (8.9% vs. 17.1% in the US), higher physical activity rates (54% vs. 50.6%), and fewer adults being overweight or obese (58.6% vs. 66.6%). Additionally, fewer individuals report heart attacks (2.8% vs. 4.2%) and strokes (2.1% vs. 3%) [18]. These state‐level patterns align with evidence from a recent systematic review and meta‐analysis showing that population‐level smoke‐free legislation is associated with significant reductions in cardiovascular events and other smoking‐related adverse outcomes [19].

CVD outcomes within states are shaped by factors such as policy development, improved insurance coverage, heightened public awareness, and lifestyle changes. Addressing these issues through targeted prevention efforts and equitable healthcare strategies is essential to reducing disparities, alongside continued research to guide effective interventions [20].

This study has several limitations. Misclassification of CVD as a cause of mortality may arise from missed or inaccurate diagnoses. Changes in coding practices across years and regions could have influenced the results. Although the AAMRs of Hawaii and Utah were similar with overlapping CIs (Hawaii: 539.4 [95% CI 536.3, 542.4], Utah: 536.6 [95% CI 533.8, 539.4]), analysis was only conducted for Utah. Underreporting of CVD on death certificates may bias estimates downward, meaning true mortality may be underestimated in certain states. Internal migration patterns, particularly movement from high‐risk rural regions to metropolitan areas, may also influence state‐level mortality trends by redistributing population risk profiles. These factors may partially attenuate or mask true disparities across states.

## 5 Conclusion

CVD‐related mortality has stabilized nationally over the past decade, with Mississippi, Oklahoma, and West Virginia showing the highest rates and Minnesota, Arizona, and Utah the lowest. The NH Black or African American population, males, and nonmetropolitan populations face the highest burden in all studied states, except in Minnesota, where the NH American Indian or Alaskan Native population experiences the highest rates. Targeted strategies addressing social and environmental risks are essential to reduce these disparities.

## Author Contributions

**Muhammad Umer Sohail:** conceptualization, methodology, investigation, data curation, formal analysis, writing – original draft, project administration. **Ruqiat Masooma Batool:** conceptualization, methodology, investigation, data curation, formal analysis, writing – original draft, visualization. **Muhammad Saad:** investigation, data curation, formal analysis, writing – review and editing. **Saad Ahmed Waqas:** investigation, data curation, writing – review and editing. **Asad Ali Ahmed Cheema:** methodology, formal analysis, supervision, writing – review and editing. **Abdul Mannan Khan Minhas:** supervision, validation, writing – review and editing, resources.

## Funding

The authors received no specific funding for this work.

## Ethics Statement

The authors have nothing to report.

## Conflicts of Interest

The authors declare no conflicts of interest.

## Data Availability

The data that support the findings of this study are available in CDC Wonder at https://wonder.cdc.gov/controller/datarequest/D77. These data were derived from the following resources available in the public domain: ‐ https://wonder.cdc.gov/, https://wonder.cdc.gov/.
